# Heritage and hesitancy: how preference for traditional Chinese medicine influences vaccine attitudes

**DOI:** 10.3389/fpubh.2024.1355720

**Published:** 2024-03-19

**Authors:** Yaxin Lan, Lei Jin

**Affiliations:** ^1^Department of Social Work, School of Sociology and Political Science, Shanghai University, Shanghai, China; ^2^Department of Sociology, The Chinese University of Hong Kong, Hong Kong SAR, China; ^3^Faculty of Social Science, The Chinese University of Hong Kong, Shatin, Hong Kong Region, Hong Kong SAR, China

**Keywords:** traditional Chinese medicine, vaccine hesitancy, complementary and alternative medicine, COVID-19 vaccine, epistemological views

## Abstract

**Introduction:**

Vaccine hesitancy, amplified by the COVID-19 pandemic, is a pressing public health challenge. This study investigates the association between Traditional Chinese Medicine (TCM) preference and COVID-19 vaccine hesitancy within China.

**Methods:**

The study uses data from the 2021 Chinese General Social Survey (CGSS) (*N* = 2,690). Logistic regressions and Karlson-Holm-Breen (KHB) method are employed to analyzed the relationship between TCM preference and vaccine hesitancy.

**Results:**

The study reaffirms prior findings by revealing a robust and stable association between TCM preference and vaccine hesitancy, which remains unaffected by socioeconomic and demographic confounders, as well as institutional trust dynamics of healthcare system. Contrary to expectations, TCM enthusiasts do not exhibit vaccine hesitancy based on divergent epistemological views concerning vaccine risks and immunity acquisition compared to biomedicine.

**Discussion:**

This research enriches understandings of the intricate relations between healthcare paradigms and vaccine attitudes, inviting further inquiry into the role of CAM in shaping vaccination behaviors across different cultures and contexts. The insights bear significant public health implications for enhancing vaccine acceptance and coverage, particularly among populations where CAM practices wield substantial influence.

## 1 Introduction

Vaccination campaigns have been pivotal in curbing epidemics and enhancing global health. However, vaccine hesitancy, defined as a delay in accepting or refusing vaccines despite their availability (1), has emerged as a significant public health challenge, particularly magnified during the COVID-19 pandemic. Research on vaccine hesitancy encompasses various approaches, including epidemiological inquiries examining the predictive effects of structural factors and social demographics (2–6), alongside policy-oriented investigations into factors influencing vaccination willingness, such as safety, effectiveness, accessibility, and institutional trust (7–10). Recent studies have also explored vaccine hesitancy through the lens of post-modern social theories, touching upon issues like neo-colonialism (11), political ideologies (12), racialism (13, 14), conspiracy theories (15, 16), and parental norms (17, 18).

An emerging area within vaccine hesitancy research is the intersection between vaccine hesitancy and Complementary and Alternative Medicine (CAM, hereafter). This area of exploration carries unique significance, primarily because vaccines, emblematic products of modern evidence-based medicine, encounter a distinct challenge when juxtaposed with the beliefs and practices of those favoring CAM as their therapeutic approach (19).

While some Western studies have addressed this aspect of vaccine hesitancy, there remains a noticeable gap in empirical research within developing countries, particularly in nations where CAM is deeply ingrained in the healthcare system. China serves as an illustrative case, with Traditional Chinese Medicine (TCM, hereafter) deeply rooted in national cultural heritage and recognized as integral to modern healthcare (20). The widespread reliance on Chinese medicine, coupled with its official inclusion in treatment guidelines during the COVID-19 pandemic (21), underscores the importance of examining how preferences for TCM influence vaccine hesitancy among Chinese populations.

This study aims to investigate how individuals’ preferences for TCM predict COVID-19 vaccine hesitancy. We seek to establish a reliable association between TCM preferences and vaccine hesitancy by controlling for a range of confounding factors, particularly satisfaction with the Chinese healthcare system. Additionally, we aim to explore whether TCM preferences influence vaccine hesitancy through distinct epistemological beliefs about vaccines and immunity, diverging from those of modern biomedicine.

In the forthcoming sections, we will first discuss the mechanisms through which CAM preferences may lead to vaccine hesitancy, proposing research hypotheses. We will then detail the data and methods. The results and research significance will follow. By investigating CAM-related factors impacting vaccine hesitancy, our study can inform more effective public health strategies and culturally sensitive healthcare policies, particularly in light of the increasing popularity of Chinese medicine.

### 1.1 Theoretical underpinnings of the relationship between CAM and vaccine hesitancy

The correlation between the preference for complementary and alternative medicine and vaccine hesitancy has recently attracted attention among researchers. Investigations into this correlation have provided valuable insights, offering a promising pathway to comprehend the multifaceted determinants of vaccine attitudes and choices. Empirical studies have unveiled diverse and intricate patterns, indicating the complexity of the association between CAM use and vaccine hesitancy (19, 22, 23). These complexities arise from several factors, including the specific practices of CAM examined in empirical studies and variations in vaccine categories. Moreover, conclusions drawn from different countries may diverge due to disparities in the importance of CAM within national healthcare systems and variations in public utilization and perceptions of CAM (24–26).

Given these disparities, it is imperative to avoid uncritically applying findings from existing studies on CAM and vaccine hesitancy to understand the situation of COVID-19 vaccine hesitancy in China, where Traditional Chinese Medicine holds widespread influence. It becomes essential to reexamine the relationship between CAM and COVID-19 vaccine hesitancy within the context of current China, grounding public health strategies on empirical and contextually relevant insights.

In light of these considerations, we propose revisiting a hypothesis previously explored in Western empirical studies within the specific context of China:

*Hypothesis 1:* In China, the preference for Traditional Chinese Medicine is associated with a higher likelihood of COVID-19 vaccine hesitancy.

### 1.2 Complexity interplay: CAM, institutional trust, and vaccine hesitancy

Prior research has consistently suggested a connection between CAM usage and vaccine hesitancy, although various factors often confound this relationship (24). One notable factor is the crisis of institutional trust, frequently cited by some CAM users when explaining their vaccine hesitancy (26–28).

CAM users tend to distrust mainstream healthcare institutions, particularly the modern medical system founded on evidence-based medicine (29). While vaccines are generally recognized as safe and effective tools for disease prevention, public skepticism can manifest in different ways, including concerns about vaccine development, production, promotion, administration, and oversight (30, 31). Some CAM users who express vaccine hesitancy may raise questions about the transparency, objectivity, and impartiality of vaccine-related processes (32). Individuals holding this viewpoint cast doubt on large pharmaceutical companies, official health institutions, and other stakeholders involved, suspecting that collective immunization campaigns are driven by profit motives and may not necessarily align with individual health priorities (33, 34).

Additionally, interactions between healthcare experts in the national medical system further exacerbate vaccine hesitancy among CAM users (32, 35). Against the backdrop of health consumerism and a culture of risk, an increasing number of individuals are advocating for greater involvement in health decision-making. They seek more autonomy and control over their health choices rather than occupying passive roles. Healthcare professionals use technical language to explain the consequences of non-vaccination, which is often met with resistance. In contrast, with their patient-centered approach, CAM practitioners tend to empower individuals with more autonomy and choice, providing them with greater control over their health decisions (25, 32).

To disentangle the influence of trust in the healthcare system from the relationship between CAM belief and vaccine hesitancy, we propose the second hypothesis:

*Hypothesis 2:* When satisfaction with the healthcare system remains constant, trust in Chinese medicine will increase the likelihood of vaccine hesitancy.

### 1.3 Epistemological differences: CAM, biomedicine, and vaccine concepts

An essential dimension influencing vaccine hesitancy among CAM users stems from fundamental epistemological disparities between CAM and biomedicine (19, 25). These disparities revolve around their distinct perspectives on health, disease, and the natural healing process (36, 37). Conventional biomedicine typically upholds double-anonymized randomized controlled trials as the pinnacle of medical evidence, prioritizing empirical validation. Conversely, CAM encompasses various ancient healing systems, such as traditional Chinese medicine, each offering unique viewpoints on the body, illness, and the effectiveness of interventions (38). For instance, qualitative studies have revealed that some CAM users prefer homeopathic immunizations over traditional vaccines, attributing greater protection to these alternatives (25).

In addressing diseases like COVID-19, vaccines are unequivocally recognized by biomedical professionals as the most effective, cost-efficient, and enduring method for prevention and control. However, TCM tends to provide a holistic approach to addressing COVID-19, perceiving it as a “cold-dampness plague” characterized by cold and damp symptoms. Based on this understanding, TCM emphasizes using Chinese herbal medicine, dietary adjustments, and external therapies to “*Wenyang*,” which translates to invigorating *Yang Qi* and restoring balance to enhance the body’s resilience (39, 40). These differing perspectives may not necessarily align with the principles governing vaccine immunization, thereby generating reservations among CAM adherents.

In light of these epistemological differences, it is essential to introduce the third hypothesis that accounts for the impact of CAM preferences on perceptions of health and disease, subsequently influencing vaccine hesitancy outcomes:

*Hypothesis 3:* Preference for Chinese medicine shapes conceptions of immunity through vaccines, subsequently impacting vaccine hesitancy outcomes.

In the upcoming sections, we will empirically test these hypotheses using data from the 2021 China General Social Survey (CGSS). We aim to gain a deeper understanding of the complex interplay between the use of TCM and vaccine hesitancy and inform public health policy within the unique sociocultural landscape of contemporary China.

## 2 Materials and methods

### 2.1 Data

This study utilizes data from the Chinese General Social Survey (CGSS), a comprehensive nationwide research project conducted by Renmin University of China. CGSS focuses on societal transitions in contemporary China, collecting data on various aspects such as population demographics, household conditions, labor and employment, social attitudes, and lifestyle. The survey employs a multi-stage stratified sampling approach, with counties serving as primary sampling units. Post-stratification weights were applied to correct oversampling, ensuring that survey results accurately represented the general population in China (41).

The data analyzed is from the 2021 wave of CGSS, conducted after China completed its nationwide vaccination campaign. CGSS 2021 includes a module documenting the impact of the COVID-19 pandemic on behavior and attitudes, alongside a detailed investigation of epidemic prevention and vaccination. Moreover, one-third of respondents answered questions from the International Social Survey Programme (ISSP) health module, which gathers additional information on healthcare quality, health beliefs, and related topics among Chinese residents.

The 2021 survey collected a total of 8,148 valid samples nationwide, with 2,690 individuals selected to respond to the ISSP health module. It should be noted that, while the data provides insights into Chinese views and behaviors regarding vaccine hesitancy and its associated factors, it is cross-sectional in nature. Despite efforts to control for confounding factors, using cross-sectional data constraints our ability to establish causality. The limitation has also been addressed in the discussion section.

### 2.2 Measures

#### 2.2.1 Vaccine hesitancy

The dependent variable, vaccine hesitancy, is operationalized through a two-step process. Respondents were first asked, “Currently, have you received the COVID-19 vaccine?” Those indicating they had not received the vaccine were further assessed based on their response to the question, “What is the main reason you do not want to receive the COVID-19 vaccine?” Individuals disqualified from vaccination due to objective conditions or lacking information on vaccine availability were excluded. The remaining population, expressing reluctance to be vaccinated due to various concerns, is considered as exhibiting vaccine hesitancy. Those identified as hesitant are coded as “1”, while others are coded as “0”.

#### 2.2.2 Preference for traditional Chinese medicine

The core independent variable is measured using a five-point Likert scale question: “To what extent do you agree that Traditional Chinese Medicine is more effective than Western medicine?” The original responses range from 1 to 5, representing “strongly agree” to “strongly disagree.” We reverse-coded the responses so that higher scores indicate greater trust in TCM, rendering it a continuous variable.

#### 2.2.3 Satisfaction with Chinese healthcare system

This variable is measured using question D20 in CGSS, which asks, “Overall, are you satisfied with China’s healthcare system?” Responses are provided on a seven-point Likert scale ranging from “1” (completely satisfied) to “7” (completely dissatisfied). We reverse-coded the answers so that higher scores indicate greater satisfaction with the Chinese healthcare system. This satisfaction score is treated as a continuous variable.

#### 2.2.4 Epistemological views on vaccination and immunity

Two statements are utilized to assess individuals’ epistemic underpinnings: “In the broader context, getting vaccinated has more disadvantages than advantages” and “It is better to acquire immunity through getting sick than through vaccination.” Respondents’ attitudes are collected using a five-point Likert scale (1–5), with higher scores indicating stronger discordance with these statements. Responses are subsequently inversed to underscore the extent of deviation from the biomedical epistemology, rendering the variable continuous in nature.

#### 2.2.5 Covariates

Control variables in our empirical analysis include gender, birth cohort, log transferred household income *per capita*, years of education, professional status, religious belief, party membership, household registration type (*hukou*), self-rated health status, marital status, cohabitation with family members, and health insurance coverage.

### 2.3 Analytical strategy

The dependent variable of this study, vaccine hesitancy, is binary. Therefore, logistic regression is employed to examine the relationship between vaccine hesitancy and its associations. Concerning potential mediations addressed in the third hypothesis, we utilize the Karlson-Holm-Breen (KHB) method (42). This technique enables us to evaluate the degree to which epistemological perspectives on vaccine immunity mediate the impact of CAM use on vaccine hesitancy, thereby offering insights into the fundamental mechanisms that underlie these associations.

Given potential regional variations in anti-pandemic measures, which may lead to prediction biases, we used the cluster standard errors so that the results can be robust to heteroscedasticity as well as autocorrelation within Chinese provinces. Sample weights provided by CGSS are utilized throughout the analysis to ensure appropriate adjustments.

## 3 Results

### 3.1 Descriptive statistics

Table 1 presents a comprehensive overview of the study variables. A total of 8,148 respondents participated in CGSS 2021, with 2,690 of them also contributing data to the ISSP module. We observe a similar distribution between the total sample and the additional module, suggesting that the inclusion of the ISSP module did not introduce significant bias into our analysis, thereby reinforcing the robustness of our findings.

**Table 1:** Descriptive statistics for study variables.

	Total	ISSP module
	(*N* = 8,148)	(*N* = 2,690)
COVID-19 Vaccine Hesitancy		
*No*	7,258 (89.2%)	2,374 (88.4%)
*Yes*	879 (10.8%)	313 (11.6%)
Preference for TCM		
Mean (SD)		3.03 (0.88)
*N* (% Missing)		2,605 (3.2%)
Satisfaction with the Healthcare System		
Mean (SD)		5.14 (1.12)
*N* (% Missing)		2,676 (0.5%)
Epistemological view on vaccine risk		
Mean (SD)		2.07 (1.02)
*N* (% Missing)		2,658 (1.2%)
Epistemological view on immunity acquisition		
Mean (SD)		2.10 (0.91)
*N* (% Missing)		2,542 (5.5%)
**Covariates**		
Household income *per capita* (log transferred)		
Mean (SD)	9.46 (2.54)	9.41 (2.57)
*N* (% Missing)	6,655 (18.3%)	2,203 (18.1%)
Years of education		
Mean (SD)	9.28 (4.65)	9.37 (4.64)
*N* (% Missing)	8,127 (0.3%)	2,682 (0.3%)
Self-rated health status		
Mean (SD)	3.48 (1.09)	3.47 (1.09)
*N* (% Missing)	8,142 (0.1%)	2,688 (0.1%)
Gender		
*Male*	3,679 (45.2%)	1,191 (44.3%)
*Female*	4,469 (54.8%)	1,499 (55.7%)
Birth cohort		
*Born before 1950*	1,089 (13.4%)	337 (12.5%)
*1950s*	1,610 (19.8%)	525 (19.5%)
*1960s*	1,661 (20.4%)	584 (21.7%)
*1970s*	1,278 (15.7%)	393 (14.6%)
*1980s*	1,171 (14.4%)	384 (14.3%)
*1990s and after*	1,339 (16.4%)	467 (17.4%)
Household registration type		
*Rural Hukou*	5,597 (69.4%)	1880 (70.7%)
*Urban Hukou*	2,464 (30.6%)	778 (29.3%)
Whether being a CPC member		
*No*	6,557 (80.6%)	2,177 (81.0%)
*Yes*	1,578 (19.4%)	509 (19.0%)
Whether being religious		
*No*	7,812 (95.9%)	2,580 (95.9%)
*Yes*	336 (4.1%)	110 (4.1%)
Current professional status		
*Not working or schooling*	3,945 (48.4%)	1,285 (47.8%)
*Working or schooling*	4,203 (51.6%)	1,405 (52.2%)
Marital Status		
*Single*	2,194 (26.9%)	713 (26.5%)
*Married*	5,954 (73.1%)	1977 (73.5%)
Whether have cohabited family member		
*No*	2,248 (27.6%)	725 (27.0%)
*Yes*	5,900 (72.4%)	1965 (73.0%)
Health insurance coverage		
*No*	407 (5.0%)	143 (5.3%)
*Yes*	7,692 (95.0%)	2,532 (94.7%)

Regarding COVID-19 vaccine hesitancy, the majority of the population had completed vaccination by the time of data collection, indicating substantial progress in achieving herd immunity in China. Specifically, 89.2% (*N* = 7,258) of participants in the total sample and 88.4% (*N* = 2,374) in the ISSP module reported no hesitancy towards vaccine acceptance. Excluding cases where vaccination was not feasible due to medical or accessibility reasons, only a small proportion (approximately 10%) of the population remained hesitant to receive the vaccine.

Participants’ preference for traditional Chinese medicine was measured on a scale with a mean score of 3.03 (SD = 0.88, ranging from 1 to 5) among those who provided data for ISSP (*N* = 2,605). It reflects a moderate level of preference for TCM among the study participants. Additionally, satisfaction with the healthcare system was assessed, yielding a mean score of 5.14 (SD = 1.12, ranging from 1 to 7) with minimal missing responses (0.5%). This score suggests a generally positive perception of the healthcare system in China.

Meanwhile, “Epistemological view on vaccine risk” records a mean score of 2.07 (SD = 1.02), while “Epistemological view on immunity acquisition” exhibits a similar mean score of 2.10 (SD = 0.91). Both of these questions employed a 1 to 5 scale for responses. The findings suggest that, on average, the respondents’ perspectives on vaccines and immunization are generally consistent with the principles of modern biomedicine.

Covariates encompass various factors, including log-transferred household income *per capita* (mean = 9.46, SD = 2.54), years of education (mean = 9.28, SD = 4.65), self-rated health status (mean = 3.48, SD = 1.09), gender distribution (45.2% male, 54.8% female), birth cohort representation, and so on. Covariates will be considered in subsequent analyses to explore their potential associations with vaccine hesitancy and TCM-related factors.

### 3.2 Correlations between TCM preferences and vaccine hesitancy

Table 2 presents the results of logistic regression models assessing the relationship between vaccine hesitancy and the preference for TCM as the primary independent variable, depicted by log odds.

**Table 2:** Logistic regressions on vaccine hesitancy and related factors (report in odds ratio).

	Model 1	Model 2	Model 3	Model 4
	*N* = 2602	*N* = 2098	*N* = 2089	*N* = 2002
Preference for TCM	1.115 (.087)	1.180* (.084)	1.187* (.085)	1.183* (.089)
Satisfaction with the Healthcare System			.881 (.061)	.880 (.064)
Epistemological view on vaccine risk				1.035 (.063)
Epistemological view on immunity acquisition				1.141 (.131)
Birth cohort (ref. born before 1950)				
*1950s*		.637* (.115)	.628* (.116)	.593** (.112)
*1960s*		.444** (.117)	.432** (.119)	.419*** (.108)
*1970s*		.358*** (.111)	.340*** (.110)	.339*** (.111)
*1980s*		.452** (.120)	.435** (.119)	.453** (.127)
*1990s and after*		.692 (.331)	.676 (.330)	.667 (.326)
Gender (ref. male)		.626** (.106)	.630** (.107)	.611** (.109)
Household income per capita		1.036 (.045)	1.037 (.045)	1.048 (.052)
Years of education		1.010 (.030)	1.007 (.030)	1.008 (.028)
Household registration type (ref. rural Hukou)		1.267 (.212)	1.247 (.212)	1.146 (.163)
CPC membership (ref. not a party member)		.727 (.159)	.730 (.158)	.730 (.160)
Religious status (ref. no religion)		1.237 (.329)	1.267 (.345)	1.236 (.330)
Professional status (ref. not working or schooling)		.741 (.119)	.733* (.114)	.722* (.113)
Marital status (ref. single)		1.268 (.356)	1.265 (.360)	1.214 (.353)
Cohabitation (ref. no cohabited family member)		.806 (.165)	.808 (.169)	.817 (.178)
Self-rated health status		.945 (.062)	.960 (.065)	.974 (.073)
Health insurance coverage (ref. no coverage)		.837 (.398)	.835 (.399)	.977 (.456)

Results are reported with estimated coefficients. Clustered robust standard errors in parentheses. * *p* < 0.05, ** *p* < 0.01, *** *p* < 0.001.

In Model 1, featuring solely the dependent variable and TCM preference, no statistically significant relationship is observed (log odds = 1.115, *p* = 0.161). However, this outcome does not warrant the rejection of the first hypothesis. Upon introducing additional covariates in Model 2, TCM preference emerges as a significant predictor of vaccine hesitancy (SE = 0.071, *p* = 0.020). When socioeconomic and sociodemographic factors are controlled, a one-unit increase in TCM preference elevates the odds of reporting vaccine hesitancy by a factor of 1.18. This finding is consistent with Hypothesis 1, indicating that a greater preference for TCM correlates with a heightened propensity towards COVID-19 vaccine hesitancy.

Furthermore, it is noteworthy that these findings are consistent with previous research, further emphasizing the significant role that CAM, including the Chinese medical paradigm, plays in shaping attitudes towards vaccine hesitancy within the population.

### 3.3 Reassessment of the confounding role of healthcare system satisfaction

In examining Hypothesis 2, Model 3 introduces satisfaction with the Chinese healthcare system. Table 2 shows that satisfaction with the healthcare system does not demonstrate a statistically significant relationship with vaccine hesitancy (SE = 0.061, *p* = 0.067). However, at this stage, the log odds for TCM preference increase to 1.187 and remain statistically significant (SE = 0.085, *p* = 0.017). In other words, even after accounting for the effect of satisfaction with the healthcare system, preference for TCM continues to influence COVID-19 vaccine hesitancy, affirming Hypothesis 2.

These findings echo the descriptive statistical outcomes presented earlier, suggesting that Chinese residents generally perceive the national healthcare system positively. In contrast to scenarios discussed in previous empirical studies, where vaccine hesitancy among CAM users may be linked to dissatisfaction with healthcare systems, this does not appear to be a significant confounding factor within the context of China.

### 3.4 Reassessment of epistemological pathways to vaccine hesitancy

In the final stage of our analysis, we examined Hypothesis 3, which posited that vaccine hesitancy among individuals who prefer TCM may stem from their distinct epistemological perspectives on vaccines and immunity, deviating from the biomedical paradigm.

We constructed Model 4 (see Table 2), incorporating epistemological variables into Model 3 to assess this hypothesis. The results (Odds ratio = 1.183, SE = 0.089, *p* = 0.025) affirm the robust association between TCM preference and vaccine hesitancy, even after adjusting for potential confounders. To delve deeper into potential mediating factors elucidating this relationship, we employed the Karlson-Holm-Breen (KHB) method (42).

Table 3 presents the results of the KHB analysis. On average, the probability of vaccine hesitancy increases by 17.57 percentage points for a standard deviation change in TCM preferences. After controlling for epistemological views, this average increase is reduced to 16.78 percentage points. However, it is worth noting that the indirect effect does not attain statistical significance (SE = 0.008, *p* = 0.309). As evidenced by Table 3, the mediating role of epistemological factors, whether related to perceptions of vaccine risk or immunity acquisition, does not significantly impact the vaccine hesitancy of TCM supporters. In fact, the mediating ratio merely accounts for 4.54% of the association between TCM preference and vaccine hesitancy.

**Table 3:** KHB decomposition for epistemological mediations.

	Total effect	Direct effect	Indirect effect	Percentage reduced
Preference for TCM	0.1757* (0.0791)	0.1678* (0.0750)		
Through epistemological view on vaccine risk			0.0006 (0.0018)	0.36%
Through epistemological view on immunity acquisition			0.0073 (0.0077)	4.18%

Results are reported with estimated coefficients. Clustered robust standard errors in parentheses. * *p* < 0.05, ** *p* < 0.01, *** *p* < 0.001.

These findings reject Hypothesis 3 and deviate to some extent from previous studies suggesting that CAM users refuse vaccination due to fundamentally different conceptions of vaccines and immunization. Our results indicate that individuals endorsing TCM may not necessarily harbor conflicting perspectives regarding vaccines and immunity vis-à-vis the biomedical standpoint. Instead, their vaccine hesitancy may be attributed to other factors, such as a preference for Chinese patent medicine or a heightened emphasis on physical exercise to prevent COVID-19.

## 4 Discussion

This study explores the influence of traditional Chinese medicine on vaccine hesitancy among the Chinese population, leveraging nationwide survey data with a substantial sample size. Our findings, situated against the backdrop of China’s response to the COVID-19 pandemic, reaffirm the well-established correlation between CAM and vaccine hesitancy. We demonstrate that endorsing TCM as a preferred medical paradigm over contemporary evidence-based Western medicine significantly predicts COVID-19 vaccine hesitancy. The study sheds light on the unique contributors to vaccine attitudes in China, offering a critical contribution to the expanding literature on CAM frameworks in the context of vaccine hesitancy. The findings will provide empirical bases for informing public health policies addressing vaccine hesitancy within China and serve as a reference for countries where indigenous medical practices profoundly influence health perceptions and behaviors.

While the results align with previous research regarding the overall association between CAM and vaccine hesitancy, we have uncovered distinctive insights within the Chinese context, prompting a reconsideration of developing tailored intervention policies. Our study reveals two notable differences: first, the role of satisfaction with the healthcare system as a confounding factor among TCM supporters’ attitudes towards vaccines, and second, the mediating effect of epistemological views regarding immunity and vaccines.

On the one hand, our findings challenge the notion that vaccine hesitancy among TCM enthusiasts mirrors the skepticism towards vaccines observed among CAM users in other contexts, often confounded by dissatisfaction with the healthcare system. Previous empirical studies across various types of vaccine hesitancy reported that CAM users often overlap with conspiracy supporters, questioning the public health recommendations from governments and authorities (29, 43). However, our study suggests otherwise. In fact, recent studies indicate that positive attitudes towards TCM’s role in combating the COVID-19 pandemic are positively associated with nationalist, patriotic, or collectivist sentiments and values (44–46). In other words, TCM supporters in China are more inclined to trust the government and national medical system, unlike CAM users elsewhere who resist vaccines due to institutional dissatisfaction. Therefore, to accurately comprehend why Chinese individuals preferring TCM exhibit vaccine hesitancy, further research is warranted to delve deeper into their political and cultural values.

Nonetheless, our result should not diminish the importance of trust in healthcare institutions as a significant predictor, as empirical evidence has revealed that regulatory failures resulting in unsafe vaccine scandals have caused anti-vaccination among Chinese parents (34). It needs to be noted that our discussion pertains to the vaccine hesitancy arising from the COVID-19 pandemic, a unique public health event characterized by substantial and mandatory interventions by the Chinese government. Correspondingly, the role of satisfaction with the healthcare system in vaccine decision-making may differ from that of routine immunizations.

On the other hand, this paper presents a counterargument to the idea that CAM users reject vaccines due to epistemological disparities. While previous studies emphasized the health worldview, attributing vaccine hesitancy among CAM supporters’ preferences on alternative wisdom emphasizing naturalness, spirituality, and intuitive understandings of health and illnesses, our study does not support this explanation. TCM and modern Western evidence-based medicine are not mutually exclusive treatment paradigms in China. Their conceptualizations and practices of vaccine immunization may even converge. Specifically, individuals preferring TCM do not necessarily perceive vaccines as ineffective for immunizing against COVID-19 but rather prioritize other therapeutic modalities from the TCM toolbox, especially when Chinese patent medications claim efficacy and are nationally promoted (45). Moreover, historical examples, such as ancient Chinese doctors’ use of prophylactic immunization against smallpox and rabies (47), suggest that TCM may have a history of engagement with vaccination practices.

In general, our findings prompt a critical reexamination of CAM as a multifaceted category, calling for a more nuanced classification and analysis. CAM encompasses diverse practices across cultures and countries, and thus, a one-size-fits-all approach to understanding its relationship with vaccine hesitancy may not suffice. TCM occupies a unique place within CAM, particularly in Asian societies, where its presence and significance far outweigh standard CAM practices in Western contexts.

To devise effective intervention policies, a deeper exploration of the intersection between modern vaccine immunization and traditional Chinese medical practices is imperative. Tailored public health campaigns are needed to address vaccine hesitancy among TCM enthusiasts, emphasizing the compatibility of TCM and vaccination or highlighting historical vaccine use within traditional Chinese medicine. Collaborative efforts between healthcare providers trained by the evidence-based medicine paradigm and TCM practitioners can amplify the efficacy of such campaigns, fostering trust and confidence in vaccination initiatives. Additionally, prioritizing health literacy and vaccine education initiatives tailored to the cultural and philosophical underpinnings of TCM preference holds promise in bridging knowledge gaps and dispelling misconceptions surrounding vaccination.

Meanwhile, in-depth profiling of the intricate social-demographic identities of Chinese TCM enthusiasts can be fundamental. For TCM supporters with certain political or cultural sentiments, leveraging national promotion campaigns to emphasize the benefits of vaccination compared to other immunization methods can be essential. This approach resonates with their ethos and may effectively bolster vaccination willingness. While the applicability of such strategies may extend beyond China to other collectivist societies where traditional medicine holds sway, caution is warranted when extrapolating findings to individualistic contexts, necessitating nuanced adaptations.

Moving forward, ongoing empirical data collection is paramount as we navigate the complexities of vaccine acceptance amidst the COVID-19 pandemic. Further research is needed to determine the generalizability of our findings to other vaccines and immunization efforts. Rigorous monitoring and assessment of intervention effectiveness will be essential for refining public health strategies and ensuring widespread vaccine acceptance in diverse cultural contexts.

The study presents several limitations. Firstly, its use of cross-sectional data limits the ability to establish causal relationships. Observed correlations between TCM preference and vaccine hesitancy may be influenced by unobservable factors such as psychological disposition or scientific literacy. Addressing causality would require more sophisticated statistical methods and longitudinal surveys. Secondly, temporal relevance can be a concern. The survey was conducted during the early stages of the COVID-19 pandemic in China when reasons for vaccine hesitancy were less complex. Given the evolving pandemic conditions and public awareness, the relationship between TCM and vaccine hesitancy may evolve over time. Lastly, the study’s focus on China may limit generalizability due to the country’s unique governmental approaches to pandemic control and potential regional specificity in vaccine hesitancy behavior. Future research can further explore this topic using longitudinal surveys and examine the influence of other structural factors, providing insights into healthcare practices in countries with diverse medical landscapes.

## 5 Conclusion

In conclusion, this study offers significant insights into the phenomenon of COVID-19 vaccine hesitancy in China. Through rigorous statistical analysis, we have demonstrated a robust link between TCM preference and vaccine hesitancy, independent of socioeconomic, demographic and healthcare institutional factors. Moreover, in Chinese context, epistemological beliefs are not the primary driver of vaccine hesitancy among TCM proponents. Moving forward, public health professionals should consider integrating traditional medical practices into modern vaccination strategies, employing culturally sensitive approaches to enhance vaccine acceptance and coverage among populations influenced by CAM paradigms. By fostering trust and collaboration with traditional healers and communities, public health initiatives could effectively tackle vaccine hesitancy and contribute to the broader goal of achieving herd immunity against pandemics.

## Data availability statement

Publicly available datasets were analyzed in this study. This data can be found at: http://www.cnsda.org/index.php?r=projects/view&id=65635422.

## Author contributions

YL: Writing – review & editing, Writing – original draft, Methodology, Formal analysis, Data curation, Conceptualization. LJ: Writing – review & editing, Supervision, Project administration, Conceptualization.

## References

[1] MacDonald NE SAGE Working Group on Vaccine Hesitancy Liang X Chaudhuri M Dube E Gellin B. Vaccine hesitancy: definition, scope and determinants. Vaccine. (2015) 33:4161–4. doi: 10.1016/j.vaccine.2015.04.036, PMID: 25896383

[2] Patzina A Dietrich H. The social gradient in COVID-19 vaccination intentions and the role of solidarity beliefs among adolescents. SSM Popul Health. (2022) 17:101054. doi: 10.1016/j.ssmph.2022.101054, PMID: 35229013 PMC8865935

[3] Takahashi S Takahashi N Sasaki S Nohara M Kawachi I. Occupational disparities in COVID-19 vaccine hesitancy in Japan. SSM Popul Health. (2022) 19:101226. doi: 10.1016/j.ssmph.2022.101226, PMID: 36119724 PMC9465492

[4] Mendolia S Walker I. COVID-19 vaccination intentions and subsequent uptake: an analysis of the role of marginalisation in society using British longitudinal data. Soc Sci Med. (2023) 321:115779. doi: 10.1016/j.socscimed.2023.115779, PMID: 36842308 PMC9930378

[5] Wu C. Racial concentration and dynamics of COVID-19 vaccination in the United States. SSM Popul Health. (2022) 19:101198. doi: 10.1016/j.ssmph.2022.101198, PMID: 35996681 PMC9387067

[6] Pronkina E Berniell I Fawaz Y Laferrère A Mira P. The COVID-19 curtain: can past communist regimes explain the vaccination divide in Europe? Soc Sci Med. (2023) 321:115759. doi: 10.1016/j.socscimed.2023.115759, PMID: 36774703 PMC9901226

[7] Mouter N de Ruijter A Ardine de Wit G Lambooij MS van Wijhe M van Exel J. “Please, you go first!” preferences for a COVID-19 vaccine among adults in the Netherlands. Soc Sci Med. (2022) 292:114626. doi: 10.1016/j.socscimed.2021.114626, PMID: 34883311 PMC8636308

[8] Callaghan T Moghtaderi A Lueck JA Hotez P Strych U Dor A. Correlates and disparities of intention to vaccinate against COVID-19. Soc Sci Med. (2021) 272:113638. doi: 10.1016/j.socscimed.2020.113638, PMID: 33414032 PMC7834845

[9] Chen JH Shiu CS. Race, ethnicity and COVID-19 vaccine concerns: a latent class analysis of data during early phase of vaccination. SSM Popul Health. (2022) 18:101073. doi: 10.1016/j.ssmph.2022.101073, PMID: 35313704 PMC8928703

[10] Verelst F Willem L Kessels R Beutels P. Individual decisions to vaccinate one’s child or oneself: a discrete choice experiment rejecting free-riding motives. Soc Sci Med. (2018) 207:106–16. doi: 10.1016/j.socscimed.2018.04.038 29738898

[11] Ojong N Agbe E. “This is most likely not the correct vaccine”: analyzing COVID-19’s viral spread and vaccine anxieties in Ghana, Cameroon, and Malawi. Soc Sci Med. (2023) 329:116001. doi: 10.1016/j.socscimed.2023.116001 37327597 PMC10229434

[12] Zhong W Broniatowski DA. Economic risk framing increases intention to vaccinate among republican COVID-19 vaccine refusers. Soc Sci Med. (2023) 317:115594. doi: 10.1016/j.socscimed.2022.115594, PMID: 36508989 PMC9726654

[13] Walter D Ophir Y Lokmanoglu AD Pruden ML. Vaccine discourse in white nationalist online communication: a mixed-methods computational approach. Soc Sci Med. (2022) 298:114859. doi: 10.1016/j.socscimed.2022.114859, PMID: 35276624

[14] Barceló J Sheen GCH Tung HH Wu WC. Vaccine nationalism among the public: a cross-country experimental evidence of own-country bias towards COVID-19 vaccination. Soc Sci Med. (2022) 310:115278. doi: 10.1016/j.socscimed.2022.115278, PMID: 35994879 PMC9376148

[15] Milošević Đorđević J Mari S Vdović M Milošević A. Links between conspiracy beliefs, vaccine knowledge, and trust: anti-vaccine behavior of Serbian adults. Soc Sci Med. (2021) 277:113930. doi: 10.1016/j.socscimed.2021.113930, PMID: 33873008 PMC8634900

[16] Stoler J Klofstad CA Enders AM Uscinski JE. Sociopolitical and psychological correlates of COVID-19 vaccine hesitancy in the United States during summer 2021. Soc Sci Med. (2022) 306:115112. doi: 10.1016/j.socscimed.2022.115112, PMID: 35700550 PMC9167731

[17] Kuan CI. Vaccine hesitancy and emerging parental norms: a qualitative study in Taiwan. Sociol Health Illn. (2022) 44:692–709. doi: 10.1111/1467-9566.13446, PMID: 35174516

[18] Peretti-Watel P Ward JK Vergelys C Bocquier A Raude J Verger P. “I think I made the right decision … I Hope I’m not wrong”. Vaccine hesitancy, commitment and trust among parents of young children. Sociol Health Illn. (2019) 41:1192–206. doi: 10.1111/1467-9566.12902, PMID: 30972804

[19] Attwell K Ward PR Meyer SB Rokkas PJ Leask J. “Do-it-yourself”: vaccine rejection and complementary and alternative medicine (CAM). Soc Sci Med. (2018) 196:106–14. doi: 10.1016/j.socscimed.2017.11.022, PMID: 29175699

[20] Dong J. The relationship between traditional Chinese medicine and modern medicine. Evid Based Complement Alternat Med. (2013) 2013:1–10. doi: 10.1155/2013/153148, PMID: 23983772 PMC3745867

[21] Li Q Wang H Li X Zheng Y Wei Y Zhang P. The role played by traditional Chinese medicine in preventing and treating COVID-19 in China. Front Med. (2020) 14:681–8. doi: 10.1007/s11684-020-0801-x, PMID: 32651936 PMC7348116

[22] Thomire A Raude J. The role of alternative and complementary medical practices in vaccine hesitancy among nurses: a cross-sectional survey in Brittany. Infect Dis Now. (2020) 51:159–63. doi: 10.1016/j.medmal.2020.09.021, PMID: 33039553

[23] Frawley JE McKenzie K Janosi J Forssman B Sullivan E Wiley K. The role of complementary and alternative medicine practitioners in the information-seeking pathway of vaccine-hesitant parents in the Blue Mountains area, Australia. Health Social Care Community. (2021) 29:e368–76. doi: 10.1111/hsc.13361, PMID: 33761160

[24] Fasce A Karlsson L Verger P Mäki O Taubert F Garrison A. Endorsement of alternative medicine and vaccine hesitancy among physicians: a cross-sectional study in four European countries. Hum Vaccin Immunother. (2023) 19:2242748. doi: 10.1080/21645515.2023.2242748, PMID: 37581343 PMC10431744

[25] Wong LP Wong PF AbuBakar S. Vaccine hesitancy and the resurgence of vaccine preventable diseases: the way forward for Malaysia, a southeast Asian country. Hum Vaccin Immunother. (2020) 16:1511–20. doi: 10.1080/21645515.2019.1706935, PMID: 31977285 PMC7482793

[26] Ward JK Gauna F Deml MJ MacKendrick N Peretti-Watel P. Diversity of attitudes towards complementary and alternative medicine (CAM) and vaccines: a representative cross-sectional study in France. Soc Sci Med. (2023) 328:115952. doi: 10.1016/J.SOCSCIMED.2023.115952, PMID: 37245262

[27] Hornsey MJ Lobera J Díaz-Catalán C. Vaccine hesitancy is strongly associated with distrust of conventional medicine, and only weakly associated with trust in alternative medicine. Soc Sci Med. (2020) 255:113019. doi: 10.1016/j.socscimed.2020.113019, PMID: 32408085

[28] Peretti-Watel P Larson HJ Ward JK Schulz WS Verger P. Vaccine hesitancy: clarifying a theoretical framework for an ambiguous notion. PLoS Curr. (2015) 7:ecurrents.outbreaks.6844c80ff9f5b273f34c91f71b7fc289. doi: 10.1371/currents.outbreaks.6844c80ff9f5b273f34c91f71b7fc289, PMID: 25789201 PMC4353679

[29] Rozbroj T Lyons A Lucke J. Psychosocial and demographic characteristics relating to vaccine attitudes in Australia. Patient Educ Couns. (2019) 102:172–9. doi: 10.1016/j.pec.2018.08.027, PMID: 30166057

[30] Jamison AM Quinn SC Freimuth VS. “You don’t trust a government vaccine”: narratives of institutional trust and influenza vaccination among African American and white adults. Soc Sci Med. (2019) 221:87–94. doi: 10.1016/j.socscimed.2018.12.020, PMID: 30576982 PMC6350921

[31] Latkin CA Dayton L Yi G Konstantopoulos A Boodram B. Trust in a COVID-19 vaccine in the U.S.: a social-ecological perspective. Soc Sci Med. (2021) 270:113684. doi: 10.1016/j.socscimed.2021.113684, PMID: 33485008 PMC7834519

[32] Ecker F Kutalek R. “I’m not an anti-vaxer!”-vaccine hesitancy among physicians: a qualitative study. Eur J Pub Health. (2021) 31:1157–63. doi: 10.1093/eurpub/ckab174, PMID: 34580713 PMC8675240

[33] Cole WM Schofer E Velasco K. Individual empowerment, institutional confidence, and vaccination rates in cross-National Perspective, 1995 to 2018. AM Sociol Rev. (2023) 88:379–417. doi: 10.1177/00031224231162869

[34] Yang R Penders B Horstman K. Addressing vaccine hesitancy in China: a scoping review of chinese scholarship. Vaccine. (2020) 8:2. doi: 10.3390/vaccines8010002 PMC7157208 31861816

[35] Deml MJ Jafflin K Merten S Huber B Buhl A Frau E. Determinants of vaccine hesitancy in Switzerland: study protocol of a mixed-methods national research programme. BMJ Open. (2019) 9:e032218. doi: 10.1136/bmjopen-2019-032218, PMID: 31678955 PMC6830664

[36] Bryden GM Browne M Rockloff M Unsworth C. Anti-vaccination and pro-CAM attitudes both reflect magical beliefs about health. Vaccine. (2018) 36:1227–34. doi: 10.1016/j.vaccine.2017.12.068, PMID: 29395527

[37] Browne M Thomson P Rockloff MJ Pennycook G. Going against the herd: psychological and cultural factors underlying the “Vaccination confidence Gap”. PLoS One. (2015) 10:e0132562. doi: 10.1371/journal.pone.0132562, PMID: 26325522 PMC4556675

[38] Deml MJ Notter J Kliem P Buhl A Huber BM Pfeiffer C. “We treat humans, not herds!”: a qualitative study of complementary and alternative medicine (CAM) providers’ individualized approaches to vaccination in Switzerland. Soc Sci Med. (2019) 240:112556. doi: 10.1016/j.socscimed.2019.112556, PMID: 31563005

[39] Zhao F Yang Z Wang N Jin K Luo Y. Traditional chinese medicine and western medicine share similar philosophical approaches to fight covid-19. Aging Dis. (2021) 12:1162–8. doi: 10.14336/AD.2021.0512, PMID: 34341699 PMC8279530

[40] Chu L Huang F Zhang M Huang B Wang Y. Current status of traditional Chinese medicine for the treatment of COVID-19 in China. Chin Med. (2021) 16:63. doi: 10.1186/s13020-021-00461-y, PMID: 34315521 PMC8314260

[41] Bian YJ Li LL. The Chinese general social survey (2003-8) sample designs and data evaluation. Chin Sociol Rev. (2012) 45:70–97. doi: 10.2753/CSA2162-0555450104

[42] Kohler U Karlson KB Holm A. Comparing coefficients of nested nonlinear probability models. Stata J. (2011) 11:420–38. doi: 10.1177/1536867x1101100306

[43] Soveri A Karlsson LC Antfolk J Lindfelt M Lewandowsky S. Unwillingness to engage in behaviors that protect against COVID-19: the role of conspiracy beliefs, trust, and endorsement of complementary and alternative medicine. BMC Public Health. (2021) 21:684. doi: 10.1186/s12889-021-10643-w, PMID: 33832446 PMC8027965

[44] Zhao H Zhang R Chen Y. The influencing role of cultural values on attitudes of the Chinese public towards traditional Chinese medicine (TCM) for the control of COVID-19. Patient Prefer Adherence. (2023) 17:3589–605. doi: 10.2147/PPA.S443713, PMID: 38169962 PMC10759415

[45] Peng AY Chen S. Traditional Chinese medicine works: a politicised scientific debate in the COVID-19 pandemic. Asian J Commun. (2021) 31:421–35. doi: 10.1080/01292986.2021.1913618

[46] Luo W Song S. Perceived benefits and barriers to Chinese COVID-19 vaccine uptake among young adults in China. Front Public Health. (2022) 10:825874. doi: 10.3389/fpubh.2022.825874, PMID: 35719675 PMC9203884

[47] Cao X. Immunology in China: the past, present and future. Nat Immunol. (2008) 9:339–42. doi: 10.1038/ni0408-339, PMID: 18349809

